# Speleotherapy – modern bio-medical perspectives

**Published:** 2014

**Authors:** H Lăzărescu, I Simionca, M Hoteteu, L Mirescu

**Affiliations:** * National Institute of Rehabilitation, Physical Medicine and Balneoclimatology, Bucharest, Romania

**Keywords:** speleotherapy, fibroblasts, salt-mine

## Abstract

Speleotherapy – a special form of climatotherapy – uses certain conditions specific to caves and salt-mines to treat several medical conditions, especially respiratory and skin-related. This reduces all types of irritations and therefore disease symptoms are mitigated or fully suppressed while the patient is accommodated into the salt-mine.

Objective: Influence of microclimate in salt-mines of Turda, Dej and Cacica on morphology and electrophoretic expression of in vitro lung and skin fibroblasts acquired from the lung and hypodermic tissues of Wistar rats, in normal conditions and after ovalbumin-induced asthma, respectively after experimental injuries and burns.

Materials and methods: skin fibroblast cultures acquired from lung and hypodermic tissue sampled from Wistar rats. Cultures acquired are developed in fibroblast monolayer attached to the culture dish. Wistar rats with weight between 75 -100 g were divided in three groups: one control group, one group with experimental asthma, one group with injuries and burns. 10 animals from each group were sent to salt-mines in Turda, Dej and Cacica for 14 days and kept in a saline environment, similar to speleotherapy.

Results: Speleotherapy applied to Wistar rats determined significant differences in cellular morphology and in electrophoretic expression of lung and skin fibroblasts from primary cultures.

Conclusions: Results of this survey indicates that speleotherapy induces changes in morphology and protein expression of in vitro lung and skin fibroblasts, and these changes support the therapeutic effects of speleotherapy.

## Introduction

Asthma is a disease characterized by the chronic inflammation of the respiratory ways, which become hyper-responsive, and also by changes in their architecture, a process referred to as remodeling. Cells responsible for the maintenance of lung structure are the parenchymatous cells of lungs, including epithelial cells, mesenchymal cells and endothelial cells. Recent surveys suggested that the function of epithelial cells, of smooth muscle cells and of fibroblasts in cultures acquired from lungs of patients with asthma differs from the function of cells cultivated in a similar manner from healthy, asthma-free individuals [**6**]. 

This survey was designed to investigate the influence of microclimate of salt-mines in Turda, Cacica and Dej on cellular morphology and the electrophoretic expression of in vitro lung fibroblasts acquired from Wistar rats, both in normal conditions and after sensitization with Ovalbumin–asthmatic rats [**1**].

Fibroblasts were cultivated from lung parenchyma sampled from control animals, from animals sensitized with ovalbumin and untreated and from rats treated in salt-mines after sensitization with Ovalbumin – placed under speleotherapy treatment. The shape of culture fibroblasts may vary depending on the substrate of growth and on the space available for movement. The use of lung fibroblast culture in the identification of therapeutic properties of salt-mine microclimate in speleotherapy treatment is a scientific method to determine the medical methodology for prevention, treatment and recovery of patients with various lung conditions [**9**].

In vitro surveys may be used to monitor cellular morphology, protein synthesis, secretion of certain chemicals, cellular metabolism, interaction of cells through cellular receptors with various ligands, capture or release of electrolytes or other types of chemicals reaching the cellular environment.

The following protocols are crucial for cellular cultures: obtaining the primary cultures, subcultivation of cells, trypsinization, blending of cellular cultures, counting of cells using the hemocytometer, assessment of cell viability: trypan blue exclusion assay, neutral red uptake assay; cytotoxicity tests: lactated hydrogenase survey, MTT assay, determination of cell growth curve, histochemical staining of senescent cells based on the activity of βgalactosidase, determination of cloning efficiency, defrost and cryoconservation of cells. Skin is the primary interface between the body and environment. The range of aggression the skin is susceptible to includes conditions caused by chemical and microbial agents, thermal and electromagnetic radiations and mechanical traumas. The consequence of skin injury is the invasion of pathogenic microorganisms that may affect human life.

The use of skin cell cultures to inspect therapeutic properties of salt-mines microclimate in speleotherapy [**5**] treatment is a scientific method to determine the medical methodology for prevention, treatment and recovery of patients with various lung conditions.

## Materials and methods

Materials: phosphate buffered saline (PBS: NaCl 0.13M + KCl 2.6mM + Na2HPO4 x12 H2O 8mM + KH2PO4 1.4mM); HAM-F12 medium (Sigma); penicillin 100 U/ml, streptomycin 100µg/ml (S.C. Antibiotice Iaşi); neomycin 50µg/ml (Sigma); fetal calf serum (Sigma).

Animal model – Wistar rats with experimental injuries and burns: Wistar rats, weights between 75-100 g, were subjected to 1 cm2 injuries and burns, on dorsal – back surface. 

Animal model – Wistar rats with allergically induced asthma: Wistar rats, weights between 75-100 g, were sensitized by using intra-muscle injections of Ovalbumin.

Primary lung fibroblasts culture

After anesthesia performed with chloroform and the sternal dissection of the animals, lung tissue was sampled and placed in phosphate buffered saline solution (PBS) on ice. Through successive trypsinizations with trypsin 0.125% and centrifugation, the cellular pellet was taken in a proper medium quantity. Cells were grown in a HAM-F12 medium with 4500 mg/l glucose, 25 mM HEPES, 100 U/ml penicillin, 100 µg/ml streptomycin and 50 µg/ml neomycin. The medium was supplemented with 10% fetal calf serum.

After 24 hours, the culture medium was replaced, to remove dead cells and cellular remains. Subsequently, the replacement of medium was performed daily. Cells were cultivated on glass Petri dishes, 50 mm diameter (Schott).

Culture of skin fibroblasts

After anesthesia performed with chloroform and the removal of hair by using sterile razor blades, a surface of 1 cm2 of skin was sampled, and placed in phosphate buffered saline solution (PBS) on ice. Through successive trypsinizations with trypsin 0.125% and centrifugation, the cellular pellet was taken in a proper medium quantity. Cells were grown in a HAM-F12 medium with 4500 mg/l glucose, 25 mM HEPES, 100 U/ml penicillin, 100 µg/ml streptomycin and 50 µg/ml neomycin. The medium was supplemented with 10% fetal calf serum. 

Phase contrast microscopy

The phase contrast microscopy, first described in 1934 by the Dutch physicist Frits Zernike, is an optical contrast technique which may be used to produce high contrast images of transparent samples, like live cells.

SDS-PAGE Electrophoresis

The electrophoresis of proteins from the total homogenous cellular lysate was destined to determine changes incurred at the level of protein expression of fibroblast cultures acquired from rats subjected to speleotherapeutic treatment.

The polyacrylamide gel electrophoresis of proteins was performed in altered conditions as per techniques described by Laemmli [**10**] (1970). Cultures were washed with PBS, scraped from the culture dish and lysed in buffer containing 0.5 M Tris- HCl, pH 6.8 + 0.05% BPB + glycerol 10% + 10% SDS. 

## Results

**Result of speleotherapy on skin cells**

The culture of the 7 days control skin cells showed a heterogeneous cellular composition made of skin fibroblasts and keratinocyte-type epithelial cells. After 7 days of culture, an advanced cellular pre-confluence stage was achieved. The cellular division had a high rate. Cellular morphology of control cells was consistent with data in specific literature.

The culture of the 7 days skin cells acquired from negative control animals, with injuries and burns and without speleotherapy treatment showed certain morphologic changes compared to control skin cells culture, noticing a significant reduction in the number of fibroblasts in culture, a reduction in the cell division rate and the exacerbation of morphopathology of culture cells. After the 7 days of culture, the pre-confluence level was much lower compared to control cases.

The culture of skin cells sampled from rats in the positive control group, without injuries and burns and subjected to speleotherapy in salt-mines of Cacica and Dej showed an intermediate stage between negative controls with injuries and burns and the control reference animal held in Biobase [**1**].

The culture of the 7 days skin cells acquired from rats with injuries and burns subjected to speleotherapy in salt-mines of Cacica and Dej showed a microscopically visible improvement of morphologic parameters, compared to negative controls. Cell density and viability was increased. 

The morphological observations were confirmed by the electrophoretic assay, proving an increase of protein expression and of the overall protein level in cell homogenate after exposure of animals with injuries and burns to saline environment of Cacica and Dej, proving reversal of morphopathological processes of culture skin cells.

**Results on lung fibroblasts**

The 7 days control culture of lung fibroblasts showed a homogenous appearance with high pre-confluence levels. The cell division reached a high rate and the cell morphology in optical microscopy was typical, as described in the specific literature.

Lung fibroblasts in the 7 days culture acquired from ovalbumin sensitized rats showed several morphological changes compared to the control culture of lung fibroblasts, noticing a reduction of the number of fibroblasts in culture, reduction of cell division rate, and also an exacerbated cell morphopathology of culture fibroblasts. After the 7 days of culture, the pre-confluence level was much lower compared to the control cases.

Lung fibroblasts in the 7 days culture acquired from ovalbumin-sensitized rats subjected to speleotherapy treatment in salt-mines of Turda, Cacica and Dej showed an improvement of morphological parameters of cells, compared to cultures acquired from asthmatic, ovalbumin sensitized, untreated rats.

Lung fibroblasts were homogenized by using Laemmli pH 6.8 buffer, and proteins from homogenates were separated by polyacrylamide gel electrophoresis with 10% SDS which maintained polypeptides in an altered state after the treatment with the strong reduction agents to eliminate secondary and tertiary structure [**10**].

The 10µl samples were loaded into the gel sample-well. One sample-well was reserved for the mixture of Sigma molecular markers of 205, 116; 97; 66; 55; 45, 36, 29, 24; 20; 14.2 and 6.5 kDa.

After the electrophoresis, the gel was colored with Coomassie Brilliant Blue R -250, which allowed the visualization of separated proteins. After the coloring, various proteins were shown as distinct ribbons in gel [**7**,**8**]. 

The analysis by using the 4th version of SynGene’s GeneTools software on each electrophoresis track allowed the cross-reference of total protein expression profiles [**11**].

Data achieved confirmed our optical microscopy observations, showing a decrease of the total protein levels to 130µg in induced asthma cases compared to 160 µg for the control case.

Speleotherapeutic treatment in the Turda salt-mine brought this parameter back to a value close to that of the control case, respectively 155 µg total proteins.

Similar data was achieved for salt-mines in Cacica and Dej. Thus, compared to the control group with a total protein level of 80,6 µg, this parameter had a value of 67.41 µg in experimentally induced asthma case, but increased to 81.95 after speleotherapy at Cacica and respectively 88.72 after the speleotherapy at Dej salt-mine [**4**].

**Comments**

This survey assessed the morphological phenotypes related to the remediation and remodeling processes of skin and lung fibroblast cultures acquired from control Wistar rats and ovalbumin-sensitized rats – a model for bronchial asthma which led to a hyper-response of the respiratory ways and a chronic remodeling of the respiratory ways, as also shown by other authors [**2**].

Compared to fibroblasts in control culture, the fibroblasts obtained from lung parenchyma of asthmatic rats and of ovalbumin-sensitized rats subjected to treatment in salt-mines of Turda, Cacica and Dej, the positive role of the saline environment in asthma treatment was proven.

Fibroblasts play a key role in the maintenance and alteration of tissue structure. The capacity of fibroblasts to migrate and proliferate in answer to chemotactic stimuli or to specific growth factors was explained by the necessity to control their accumulation in areas where tissue repair occurred. The capacity of fibroblasts to produce and remodel extracellular matrix contributed to the structural changes in tissues.

This survey supports the idea that phenotypically altered fibroblasts may contribute to the remodeling of the respiratory tract within asthmatic conditions. Fibroblasts cultivated from lungs of chronically ovalbumin-sensitized animals proved a constant increase of reparatory responses [**12**].

## Conclusions

The findings related to cellular morphology were confirmed by the electrophoretic assay, which proved – based on alteration of profile of several proteins and on determination of the total protein level – that exposure to environment of salt-mines in Turda, Cacica and Dej favor the in vitro acquisition of FIBRO cells and lung a fibroblasts [**3**];

Ovalbumin-sensitization of lab animals significantly decreased the number of skin cells and lung fibroblasts in cultures, and increased the morphopathological level [**3**].
